# Evaluation of the Association between Maternal Smoking, Childhood Obesity, and Metabolic Disorders: A National Toxicology Program Workshop Review

**DOI:** 10.1289/ehp.1205404

**Published:** 2012-12-11

**Authors:** Mamta Behl, Deepa Rao, Kjersti Aagaard, Terry L. Davidson, Edward D. Levin, Theodore A. Slotkin, Supriya Srinivasan, David Wallinga, Morris F. White, Vickie R. Walker, Kristina A. Thayer, Alison C. Holloway

**Affiliations:** 1 Kelly Government Solutions, Research Triangle Park, North Carolina, USA; 2 Division of National Toxicology Program, National Institute of Environmental Sciences (NIEHS), National Institutes of Health (NIH), Department of Health and Human Services (DHHS), Research Triangle Park, North Carolina, USA; 3 Integrated Laboratory Systems Inc., Research Triangle Park, North Carolina, USA; 4 Department of Obstetrics and Gynecology, Baylor College of Medicine, Houston, Texas, USA; 5 Department of Psychological Sciences, Purdue University, West Lafayette, Indiana, USA; 6 Department of Psychiatry and Behavioral Sciences, Duke University Medical Center, Durham, North Carolina, USA; 7 Department of Pharmacology and Cancer Biology, Duke University, Durham, North Carolina, USA; 8 Department of Chemical Physiology, The Scripps Research Institute, La Jolla, California, USA; 9 Food and Health Program, Institute for Agriculture and Trade Policy, Minneapolis, Minnesota, USA; 10 Howard Hughes Medical Institute, Division of Endocrinology, Children’s Hospital Boston, Boston, Massachusetts, USA; 11 Division of National Toxicology Program, Office of Health Assessment and Translation, NIEHS, NIH, DHHS, Research Triangle Park, North Carolina, USA; 12 Reproductive Biology Division, Department of Obstetrics and Gynecology, McMaster University, Hamilton, Ontario, Canada

**Keywords:** animal, chemically induced/epidemiology, diabetes, environmental epidemiology, glucose, insulin, maternal smoking toxicity, metabolism, nicotine toxicity, obesity

## Abstract

Background: An emerging literature suggests that environmental chemicals may play a role in the development of childhood obesity and metabolic disorders, especially when exposure occurs early in life.

Objective: Here we assess the association between these health outcomes and exposure to maternal smoking during pregnancy as part of a broader effort to develop a research agenda to better understand the role of environmental chemicals as potential risk factors for obesity and metabolic disorders.

Methods: PubMed was searched up to 8 March 2012 for epidemiological and experimental animal studies related to maternal smoking or nicotine exposure during pregnancy and childhood obesity or metabolic disorders at any age. A total of 101 studies—83 in humans and 18 in animals—were identified as the primary literature.

Discussion: Current epidemiological data support a positive association between maternal smoking and increased risk of obesity or overweight in offspring. The data strongly suggest a causal relation, although the possibility that the association is attributable to unmeasured residual confounding cannot be completely ruled out. This conclusion is supported by findings from laboratory animals exposed to nicotine during development. The existing literature on human exposures does not support an association between maternal smoking during pregnancy and type 1 diabetes in offspring. Too few human studies have assessed outcomes related to type 2 diabetes or metabolic syndrome to reach conclusions based on patterns of findings. There may be a number of mechanistic pathways important for the development of aberrant metabolic outcomes following perinatal exposure to cigarette smoke, which remain largely unexplored.

Conclusions: From a toxicological perspective, the linkages between maternal smoking during pregnancy and childhood overweight/obesity provide proof-of-concept of how early-life exposure to an environmental toxicant can be a risk factor for childhood obesity.

Childhood obesity and diabetes are major threats to public health in the United States and abroad. In the United States, the prevalence of obesity among children and adolescents has almost tripled since 1980, with an estimated 16.9% of children and adolescents (i.e., approximately 12.5 million children and adolescents) considered obese (Ogden and Carroll 2010). This trend is also apparent in preschool children 2–5 years of age, where obesity was shown to have increased from 5% in 1976–1980 to 10.4% in 2007–2008 (Ogden and Carroll 2010). Kim et al. (2006) reported an almost doubling in the prevalence of overweight in infants 0–6 months of age from 1980 to 2001 (3.4% to 5.9%) based on information from well-child visits at a large HMO (health maintenance organization) in Massachusetts. Understanding the factors that contribute to the childhood obesity epidemic in order to identify intervention strategies is a critical public health need, given that obesity is a known risk factor for diabetes and other chronic conditions such as heart disease (Reilly and Kelly 2011). Diabetes incidence, both type 1 (T1D) and type 2 (T2D), is also on the rise [DIAMOND Project Group 2006; Centers for Disease Control and Prevention (CDC) 2011; Dahlquist et al. 2011; Patterson et al. 2009]. Based on data from the period 2005–2008, 25.6 million (11.3%) of all people in the United States ≥ 20 years of age have diagnosed or undiagnosed diabetes (CDC 2011). Although it is estimated that 70% of T2D can be attributed to being overweight or obese (Eyre et al. 2004), 30% of cases are not attributable to obesity. Therefore, factors other than changes in physical activity or diet may be contributing to this rise in childhood obesity and metabolic disorders. Indeed, there is growing concern that perinatal exposure to chemical insults may play a significant role in the increased incidence of obesity and metabolic disorders including diabetes, possibly through direct “diabetogenic” effects or by acting as “obesogens.”

Research addressing the role of environmental chemicals in childhood obesity and diabetes has rapidly expanded in the past several years. The White House Task Force on Childhood Obesity (2010), the National Institutes of Health (NIH) Obesity Research Task Force (2011), and the Diabetes Strategic Plan from the National Institute of Diabetes and Digestive and Kidney Diseases (NIDDK 2011) all acknowledge the growing science base in this area and cite the need to understand more about the role of environmental exposures as part of future research and prevention strategies.

To help develop such a research strategy, the National Toxicology Program organized a state-of-the-science workshop in January 2011 titled “Role of Environmental Chemicals in the Development of Diabetes and Obesity” (National Toxicology Program 2011). This review derives from discussions that occurred during that workshop.

## Identification of Relevant Studies

A PubMed (http://www.ncbi.nlm.nih.gov/pubmed) search strategy updated through 8 March 2012 was developed to identify human and animal studies related to maternal smoking or tobacco exposure during pregnancy and *a*) weight, growth, or adiposity after infancy (≥ 1 year of age in humans, after postnatal day 21 in rodents), or *b*) health outcomes in offspring at any age related to T1D or T2D, glycemic control, or metabolic syndrome. Studies assessing growth in infants < 1 year of age were excluded because maternal smoking during pregnancy is a known risk factor for low birth weight, whereas the primary question being addressed at the workshop was whether maternal smoking is a risk factor for childhood obesity. The literature search included both MeSH-based and key word strategies to identify articles not yet indexed in PubMed [see Supplemental Material for a complete list of search terms (http://dx.doi.org/10.1289/ehp.1205404)]. This search identified a total of 1,551 publications (see Supplemental Material, Figure S1). Of these, 99 studies presented original data that met our inclusion criteria and were considered relevant as the primary literature. Two additional epidemiological studies identified during the peer-review process were also included (Fasting et al. 2009; Mendez et al. 2008) for a total of 101 studies (83 human studies and 18 animal studies).

## Maternal Smoking during Pregnancy

**Figure 1:**
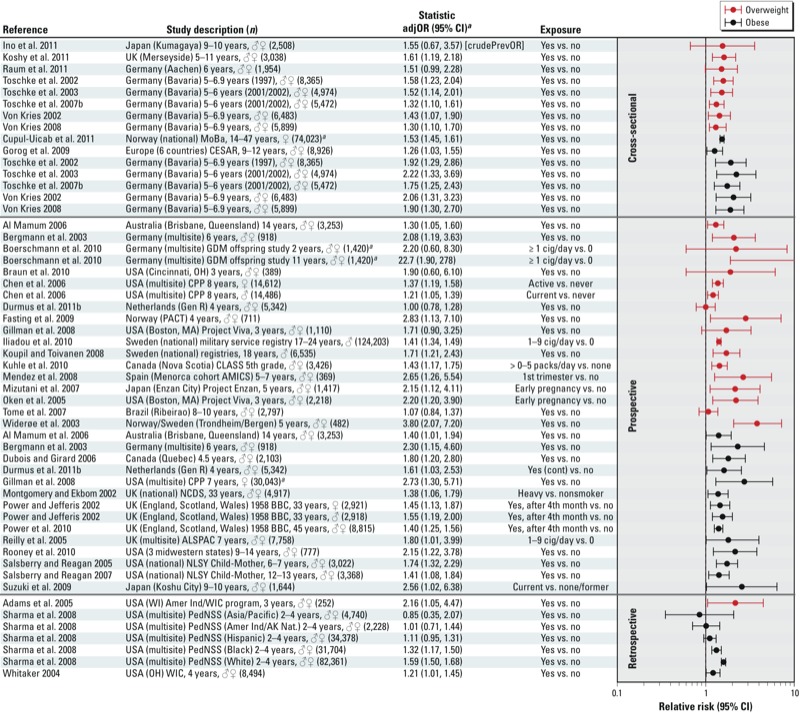
Human studies on maternal smoking during pregnancy and childhood overweight and obesity. The primary grouping of studies is based on study design [cross-sectional, prospective, or retrospective]. Within each study design, main findings are grouped by whether the outcome was overweight or obesity. Studies are then sorted alphabetically within these grouping categories. Abbreviations: AK Nat, Alaska Native; ALSPAC, Avon Longitudinal Study of Parents and Children; Amer Ind, American Indian; AMICS, Asthma Multicenter Infant Cohort Study; BBC, British Birth Cohort; CESAR, Central European Study on Air Pollution and Respiratory Health; cig, cigarettes; CLASS, Children’s Lifestyle and School Performance study; cont, continuous; CPP, Collaborative Perinatal Project; GDM, gestational diabetes mellitus; Gen R, Generation R Study; NCDS, National Child Development Study; NLSY, National Longitudinal Survey of Youth; PACT, Prevention of Allergy among Children of Trondheim study; PedNSS, Pediatric Nutrition Surveillance System; PrevOR, prevalence ratio; WIC, Women, Infants, and Children program. ***^a^***Relative risk estimates for bracketed statistics, i.e., [crudePrevOR], calculated based on data presented in the paper using an open source epidemiology statistics programs, OpenEpi (http://www.openepi.com/menu/openEpiMenu.htm).

*Offspring overweight or obesity.* Smoking during pregnancy is a known risk factor for low birth weight and small-for-gestational-age infants (Lumley et al. 2008; Oken et al. 2008; Samper et al. 2011), but is increasingly accepted as a risk factor for childhood overweight and obesity based on the consistent positive associations reported among studies (Figure 1). Most studies (34 of 42) summarized in Figure 1 present results that support a causal link between maternal smoking and subsequent child overweight or obesity. Many of these studies were evaluated in two recent meta-analyses (Ino 2010; Oken et al. 2008). Based on results reported in 14 observational studies, maternal smoking during pregnancy was associated with overweight at 3–33 years of age [pooled adjusted odds ratio (adjOR) = 1.50; 95% CI: 1.36, 1.65] (Oken et al. 2008). The pooled OR estimated by Ino (2010) for obesity [body mass index (BMI) > 95th percentile] was 1.64 (95% CI: 1.42, 1.90) based on 16 studies. Women who smoked during pregnancy tended to weigh more, and tended to have lower socioeconomic status, have less education, and were less likely to breastfeed (Ino 2010; Oken et al. 2008). Both meta-analyses used funnel plot methods to ascertain publication bias and concluded there was some evidence for publication bias, but not enough to negate the overall conclusion of increased risk.

*Ethnicity may affect the association between maternal smoking and childhood obesity.* A large retrospective study by Sharma et al. (2008) of the PedNSS (Pediatric Nutrition Surveillance System) cohort found significant associations between maternal smoking and childhood obesity only in white (*n* = 66,191) and black (*n* = 28,230) children; the associations were not apparent for Hispanic (*n* = 34,378), American Indian/Alaska native (*n* = 2,228), or Asian/Pacific Islander children (*n* = 4,740). The lack of association in children of Asian/Pacific Island descent is interesting because studies on Japanese children have reported findings consistent with a positive relationship (Ino et al. 2011; Mizutani et al. 2007; Suzuki et al. 2009, 2011). Too few studies are available on Native American and Hispanic children to interpret the findings of Sharma et al. (2008) in the context of other studies. One Brazilian study by Tome et al. (2007) did not find an association between maternal smoking and offspring overweight at 8–10 years of age (adjOR = 1.07; 95% CI: 0.84, 1.37); however, they did find that children of mothers who smoked were less likely to be underweight (adjOR = 0.56; 95% CI: 0.40, 0.78). It is worth noting that this study was conducted in the Ribeirao region of Brazil where 9.5% of the children were malnourished (BMI < 5th percentile).

*Unmeasured confounding.* Although the literature on the association between maternal smoking and obesity/overweight in children is fairly consistent when looking across studies, there are some indications that unmeasured familial or lifestyle factors may contribute to the association. One study conducted sibling-based analyses to evaluate the relationship between maternal smoking and BMI in 124,203 males born between 1983 and 1988, identified in the Swedish Medical Birth Registry with the intent of investigating the impact of familial factors on the association between maternal smoking and child obesity (Iliadou et al. 2010). They reported an association between maternal smoking during the first trimester and overweight (BMI ≥ 25) for the cohort as a whole (adjOR = 1.56; 95% CI: 1.46, 1.66 for > 10 cigarettes/day compared with 0 cigarettes). However, the authors found smaller associations based on within-family analyses, suggesting partial confounding by familial factors. Gilman et al. (2008) also conducted a sibling-based analysis of children enrolled in the Collaborative Perinatal Project to address the issue of unmeasured confounding factors by fitting conditional fixed-effects models among siblings. This type of analysis capitalizes on variability in exposure to tobacco smoke that can occur among siblings when the mother’s smoking pattern during gestation differed across pregnancies. Conceptually, the sibling analysis matches exposed and unexposed siblings on family background to identify evidence of unmeasured sources of confounding. Gilman et al. (2008) found significant positive associations in the full cohort for overweight at 7 years of age were still apparent in the conditional sibling fixed effects analysis.

*Secondhand smoke exposure during pregnancy or childhood.* Although the literature search strategy and inclusion criteria were constructed to identify studies assessing maternal smoking during pregnancy, several studies of paternal or “partner” smoking during pregnancy (Durmus et al. 2011b; Kwok et al. 2010; Leary et al. 2006; Matijasevich et al. 2011) or during childhood (von Kries et al. 2008) were identified. Some of these studies reported no association between parental/partner smoking and BMI or adiposity end points (Durmus et al. 2011b; Matijasevich et al. 2011) whereas others did (Kwok et al. 2010; Leary et al. 2006; von Kries et al. 2008), suggesting that paternal/partner smoking may be a contributing factor on its own, either by unmeasured familial factors, or as a contributor to maternal exposure from secondhand smoke. Another study by Steur et al. (2010) assessed predictors of obesity at 8 years of age in 1,687 children participating in the Prevention and Incidence of Asthma and Mite Allergy (PIAMA) study, a prospective study based in the Netherlands. Maternal smoking during pregnancy and “smoking in the parental house” were both included in the model selection, but only “smoking in the parental house” was selected as a significant independent predictor of childhood overweight.

*Other measures of adiposity, BMI, and leptin*. The findings discussed above focus on studies of associations between maternal smoking and overweight or obesity. Other studies have estimated associations between maternal smoking during pregnancy and continuous measures of BMI, standardized BMI (i.e., *z*-score or SD score), or body weight in the offspring [see Supplemental Material, Table S1 (http://dx.doi.org/10.1289/ehp.1205404)] (Beyerlein et al. 2010, 2011; Durmus et al. 2011a; Goldani et al. 2007; Hill et al. 2005; Iliadou et al. 2010; Kanellopoulos et al. 2007; Leary et al. 2006; Matijasevich et al. 2011; Ravnborg et al. 2011; Suzuki et al. 2011; Syme et al. 2010). Overall, these studies have reported positive associations with maternal smoking during pregnancy and child BMI. There are also indications that the children of mothers who smoked during pregnancy display more rapid weight gain earlier in life (Griffiths et al. 2010; Karaolis-Danckert et al. 2008; Wen et al. 2011).

Studies in the literature often assess obesity based on BMI, which is not necessarily a direct measure of adiposity because it does not distinguish fat and lean mass (Freedman and Sherry 2009; Wells 2001). Associations between maternal smoking and indicators of adiposity (i.e., body fat, abdominal fat, skin fold thickness) [see Supplemental Material, Table S2 (http://dx.doi.org/10.1289/ehp.1205404); Durmus et al. 2011a; Leary et al. 2006; Silva et al. 2010; Syme et al. 2010; Vik et al. 1996; von Schnurbein et al. 2011] are less consistent than associations with overweight/obesity or continuous measures of BMI-related end points (Figure 1). Given the limitations of BMI as a measure of adiposity, it is therefore important for future studies to consider other more direct measures of adiposity/fat mass to clarify the contribution of maternal smoking to childhood obesity.

Leptin, a protein produced by adipocytes and placental tissue, appears to function as a link between adiposity, satiety, and energy regulation (Lee and Fried 2009; Pelleymounter et al. 1995; Woods and D’Alessio 2008). Leptin levels have been correlated with neonatal growth (Christou et al. 2001; Vatten et al. 2002), but very few studies have looked at leptin levels beyond the neonatal period, and it is unclear how informative the infant studies are for addressing issues related to childhood obesity, given the complex role that maternal smoking has on childhood growth (i.e., as a risk factor for low birth weight and for childhood obesity). Overall, the findings on leptin levels in cord blood or blood collected from infants in association with maternal smoking status are mixed, with some studies reporting positive associations (Helland et al. 2001), negative associations (Mantzoros et al. 1997; Ozkan et al. 2005), or no association (Coutant et al. 2001; Kayemba-Kay’s et al. 2008; Pardo et al. 2004; Vatten et al. 2002).

**Figure 2:**
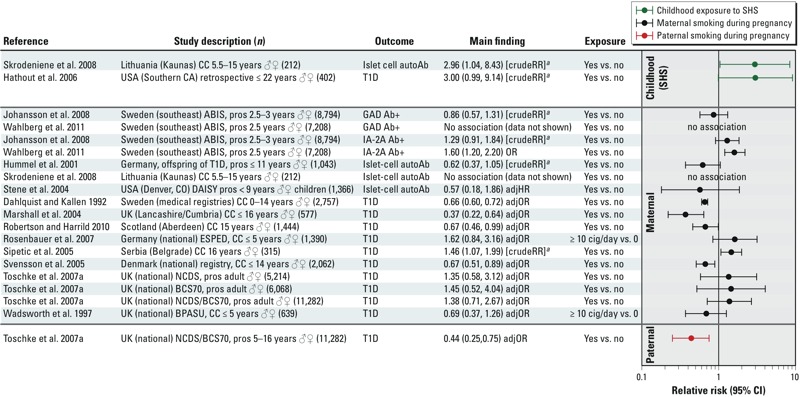
Human studies on exposure to smoking during pregnancy related to T1D. Studies are grouped by the nature of the smoke exposure (maternal, paternal, or secondhand smoke during childhood). Studies are then sorted by specific outcome measure (e.g., GAD Ab+), and presented alphabetically within a given outcome measure. Abbreviations: ABIS, All Babies in Southeast Sweden cohort; adjHR, adjusted hazard ratio; autoAb, auto antibodies; BBC, British Birth Cohort; BCS70, 1970 British Birth Cohort study; BPASU, British Paediatric Association Surveillance Unit; CC, case–control; cross-sect, cross-sectional; DAISY, Diabetes Autoimmunity Study in the Young; ESPED, German pediatric surveillance unit; GADA, glutamic acid decarboxylase antibodies; IA-2A, insulinoma antigen 2A; NCDS, National Child Development Study; pros, prospective; retro, retrospective; SHS, secondhand smoke. ***^a^***Relative risk estimates [crudeRR] calculated based on data presented in the paper using an open source epidemiology statistics programs, OpenEpi (http://www.openepi.com/menu/openEpiMenu.htm).

*T1D.* The existing human studies do not provide support for an association between maternal or paternal smoking during pregnancy and childhood T1D (Figure 2). Only 2 of the 13 studies assessing end points related to T1D following exposure during pregnancy reported findings consistent with an association (Sipetic et al. 2005; Wahlberg et al. 2011). In the study by Sipetic et al. (2005), the frequency of maternal smoking during pregnancy was higher in T1D cases compared with controls (37.2% vs. 24.8%, *p* = 0.023 or unadjusted OR = 1.79; 95% CI: 1.08, 2.97). Although the univariate finding was statistically significant, the authors did not include maternal smoking in the multivariate analysis conducted to identify risk factors for T1D. In the study by Wahlberg et al. (2011), a positive association was observed for one indicator of β-cell autoimmunity in T1D, tyrosine phosphatase (IA-2A) + antibody status (unadjusted OR = 1.6; 95% CI: 1.2, 2.2), and not for glutamic acid decarboxylase (GAD) + antibody status (data not shown). None of the five prospective studies reported a positive association between maternal or paternal smoking during pregnancy and T1D (Hummel et al. 2001; Johansson et al. 2008; Rosenbauer et al. 2007; Stene et al. 2004; Toschke et al. 2007a; Wadsworth et al. 1997), including two studies of children who were considered at high risk for developing the disease (Hummel et al. 2001; Stene et al. 2004). Several studies reported that T1D was less likely among children whose mothers smoked during pregnancy than among the children of nonsmoking mothers (Dahlquist and Kallen 1992; Marshall et al. 2004; Svensson et al. 2005) for T1D. Hummel et al. (2001) reported a nonsignificant negative association between islet-cell antibodies (a marker of β-cell autoimmunity) and maternal smoking during pregnancy among children considered at high risk because one or both parents had T1D. However, the number of cases in this study was small (47 of 1,043 children). One hypothesis that may explain evidence of a protective effect of exposure to smoking relates to the hygiene theory of autoimmune disease; this suggests that the risk of autoimmune disease may be increased if the environment is “cleaner,” which might be manifest in the households of nonsmokers (Johansson et al. 2008; Marshall et al. 2004). Alternatively, smoking may affect immune function, resulting in a reduced risk of developing autoimmune disorder (Prescott 2008).

Although the overall literature does not support an association between maternal smoking during pregnancy and T1D in offspring, associations with exposure to smoke during childhood should be investigated further. Three studies have assessed childhood exposure and T1D, and both reported findings supportive of an association (Hathout et al. 2006; Sipetic et al. 2005; Skrodeniene et al. 2008). In the study by Skrodeniene et al. (2008), Lithuanian children who were islet-cell autoantibody positive were more likely to live in a home where family members smoked indoors than children who were islet-cell autoantibody negative [7 of 13 (53.8%) vs. 53 of 199 (26.6%), respectively, *p* = 0.004; unadjusted OR = 3.21; 95% CI: 1.03, 10.00]. The frequency of mothers who reported smoking during pregnancy did not differ between the groups in this study. Hathout et al. (2006) reported that children with T1D were more likely to have been exposed to passive smoking than control children in Southern California [31 of 102 cases (30%) vs. 3 of 10 controls (10%), *p* = 0.0001; unadjusted OR = 3.90; 95% CI: 1.19, 17.24]. One limitation in the existing literature that needs to be addressed in future studies investigating the association of smoking or other environmental exposures and T1D is use of statistical models that consider potential confounding factors (e.g., viruses, nutrition, or socioeconomic psychosocial factors).

**Figure 3:**
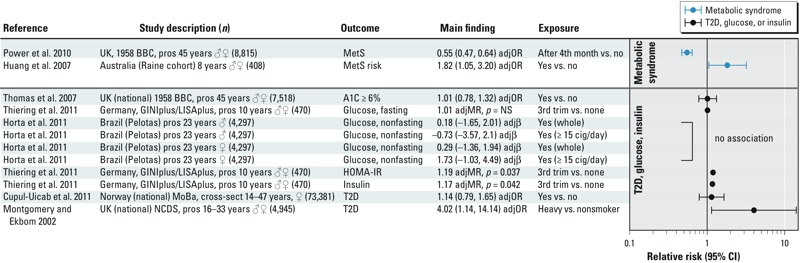
Human studies on exposure to smoking during pregnancy and findings related to T2D or metabolic syndrome. The primary grouping is whether the outcome was related to T2D, glucose, or insulin or metabolic syndrome. Studies are then sorted by specific outcome measure (e.g., HOMA-IR) and presented alphabetically within a given outcome measure. Abbreviations: A1C, glycosylated haemoglobin A1c; adjMR, adjusted mean ratio; BBC, British Birth Cohort; cig, cigarettes; cross-sect, cross-sectional; GINIplus, German Infant Nutritional Intervention study; HOMA-IR, Homeostatic model assessment for insulin resistance; LISAplus, Lifestyle-Related Factors on the Immune System and the Development of Allergies in Childhood study; MoBa, Norwegian Mother and Child Cohort Study; NCDS, National Child Development Study; NS, not significant; pros, prospective; trim, trimester.

*T2D and metabolic syndrome*. Fewer studies have looked at associations between maternal smoking during pregnancy and T2D, blood glucose, blood insulin, or metabolic disease (Horta et al. 2011; Huang et al. 2007; Montgomery and Ekbom 2002; Thiering et al. 2011; Thomas et al. 2007) (Figure 3). It is not possible to reach conclusions with confidence based on the existing literature because of the variation in health outcomes assessed among the studies [i.e., HbA1C (hemoglobin A1C); fasting glucose, nonfasting glucose, HOMA-IR (homeostatic model assessment–insulin resistance), blood insulin; T2D; and metabolic syndrome. In addition, although most of the studies do not report positive associations with these outcomes, the age at assessment in many of the studies was relatively young, and additional follow-up may be required. It is worth noting that no association with HbA1C ≥ 6% was found in the oldest and largest cohort assessed [7,518 men and women from the 1958 British birth cohort enrolled in the Perinatal Mortality Survey (PMS) evaluated at 45 years of age] (Thomas et al. 2007). This finding does not confirm an earlier report for the same cohort of an association between heavy smoking during pregnancy and T2D in offspring at 33 years of age (adjOR = 4.02; 95% CI: 1.14, 14.14) (Montgomery and Ekbom 2002). A related analysis of the cohort by Power et al. (2010) also reported no association with HbA1C in adults at 45 years of age (fully adjusted mean difference = 0.13; 95% CI: –0.33, 0.58). Power et al. (2010) reported a positive association between maternal smoking during pregnancy and metabolic syndrome based on an unadjusted model [unadjusted OR = 1.21; 95% CI: 1.05, 1.39), but there was a significant negative association between maternal smoking and metabolic syndrome after adjustment for adult life covariates such as social class, education, physical activity and inactivity (television/computer use), smoking, and consumption of fruit/vegetables, cakes/sweets, and alcohol (adjOR = 0.55; 95% CI: 0.47, 0.64).

## Animal Studies on Developmental Exposure to Nicotine

There are 599 known cigarette additives (Rabinoff et al. 2007), and most are uncharacterized for potential toxicity, including metabolic effects. One exception is cadmium, a constituent of tobacco of smoke shown to alter glucose homeostasis or insulin sensitivity in animals exposed as adults (Edwards and Prozialeck 2009) but has not been assessed for metabolic effects after developmental exposure. Only two studies assessed prenatal exposure to cigarette smoke during pregnancy and body weight later in life in offspring (Chen et al. 2011; Ng et al. 2009).

**Figure 4:**
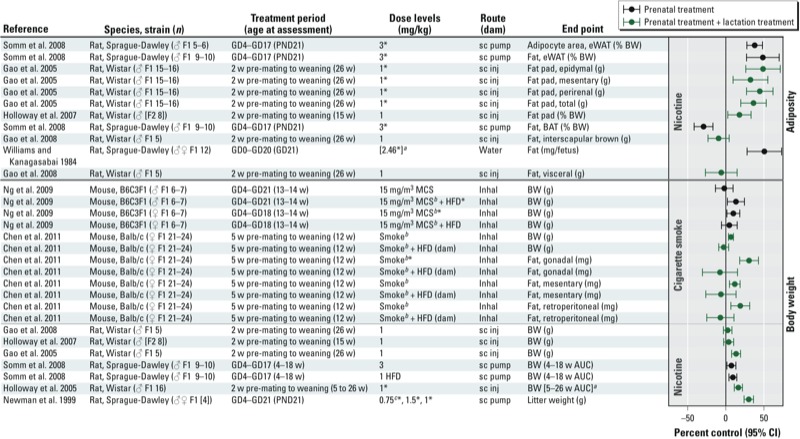
Animal studies of prenatal or prenatal + lactational exposure to nicotine or cigarette smoking and adiposity-related end points. The primary grouping of studies is based on whether the end point was related to adiposity or body weight. Within each end point category, main findings are grouped by whether the exposure was to nicotine or cigarette smoke. Within each exposure category, main findings were sorted based on specific end point (A to Z). Abbreviations: AUC, area under the curve; BAT, brown adipose tissue; BW, body weight; eWAT, epididymal white adipose tissue; GD, gestational day; inhal, inhalation; inj, injection; MCS, mainstream cigarette smoke; PND, postnatal day; sc, subcutaneous; w, weeks. ***^a^***Value was assumed or estimated based on data presented in publication. ***^b^***In Ng et al. (2009) animals were exposed via whole body inhalation to mainstream cigarette smoke (smoke inhaled by an active smoker) at a particle concentration of 15 mg/m^3^; in Chen et al. (2011) dams were exposed to exposed to cigarette smoke via a perspex chamber for 30 min 5 days/week (2 cigarettes/day, nicotine ≤ 1.2 mg, carbon monoxide ≤ 15 mg). ***^c^***Dose level summarized in graph. *Statistically significant effect at specified dose level as reported in publication. In some cases statistical significance of percent control response differs from author’s interpretation (e.g., author’s statistical analysis considered multiple comparisons, i.e., analysis of variance).

*Body weight and adiposity*. Most animal studies have reported that rats exposed to nicotine during perinatal development tended to have a higher body weight and more fat mass compared with controls (Figure 4), with the effect typically first apparent at weaning and persisting through adulthood (Gao et al. 2005; Newman et al. 1999; Oliveira et al. 2009, 2010; Somm et al. 2008). However, there are exceptions to this pattern (Gao et al. 2008; Holloway et al. 2007), which do not appear to be attributable to strain differences, dosing, or the timing of exposure. Food intake was unaffected in studies that evaluated it as an end point (Oliveira et al. 2009, 2010; Somm et al. 2008). The potential interaction between perinatal nicotine exposure and postnatal diet has been explored in only one study, which examined whether a high-fat diet (HFD) postnatally would exacerbate the weight gain in nicotine-exposed animals (Somm et al. 2008). Although the nicotine-treated animals that were fed an HFD did not consume more kilocalories than controls fed the same diet, they were heavier. The nicotine-treated animals were less physically active than control animals that consumed the same diet. There were no differences in oxygen consumption or respiratory exchange ratio, suggesting that increased weight gain in HFD animals with the same caloric intake was perhaps attributable to the dysregulation of adipose tissue development, thereby leading to higher amounts of fat storage.

**Figure 5:**
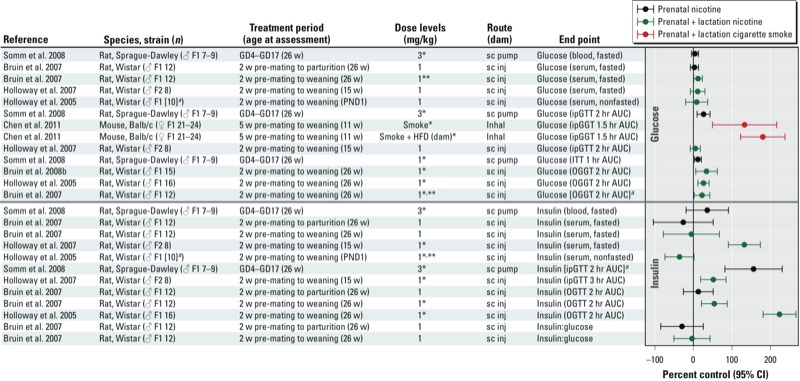
Animal studies of prenatal or prenatal + lactational exposure to nicotine and glucose homeostasis-related end points. The primary grouping of studies is based on whether the end point was based on glucose or insulin. Within the end point category, main findings were sorted based on specific end point (A to Z). Abbreviations: AUC, area under the curve; GD, gestational day; ipGTT, intraperitoneal glucose tolerance test; inj, injection; OGTT, oral glucose tolerance test; PND, postnatal day; sc, subcutaneous; w, weeks. ***^a^***Value was assumed or estimated based on data presented in publication. *Statistically significant effect at specified dose level as reported in publication. **In some cases statistical significance of percent control response differs from author’s interpretation (e.g., author’s statistical analysis considered multiple comparisons, i.e., analysis of variance).

*Glucose homeostasis and insulin sensitivity.* There are several reports in the literature on impaired glucose homeostasis in the male offspring of rats that were treated with nicotine during gestation (Somm et al. 2008), lactation (Oliveira et al. 2010), or gestation and lactation (Bruin et al. 2007, 2008c; Holloway et al. 2005) (Figure 5). Administered doses ranged from 1 to 6 mg/kg-day delivered to the mother either via an osmotic minipump implanted under the skin or via daily subcutaneous injections. These dosing protocols resulted in maternal serum cotinine levels considered relevant to women who smoke or use nicotine patches as cigarette substitutes (Bruin et al. 2007; Hackman et al. 1999; Somm et al. 2009). The most consistent findings from these studies were indications of insulin resistance in adulthood based on increased insulin area under the curve (AUC) following an oral or intraperitoneal glucose challenge (Bruin et al. 2007, 2008c; Holloway et al. 2005; Somm et al. 2008) or on an increased insulin resistance index (Oliveira et al. 2010). These effects were seen across studies despite the use of different doses of nicotine, different administration protocols, and different windows of developmental exposure within the perinatal period (i.e., gestation and/or lactation). A study by Holloway et al. (2007), reported increased insulin resistance in 15-week-old F2 generation male offspring of dams that were treated with nicotine only during gestation and lactation compared with untreated controls. Impairments in glucose tolerance also have been reported following nicotine exposure (Bruin et al. 2007, 2008c; Holloway et al. 2005; Somm et al. 2008). Effects on fasting insulin levels have been less consistent, with some studies showing no effect (Bruin et al. 2007; Somm et al. 2008) and others reporting increased levels in adult rodents compared with untreated controls (Holloway et al. 2005, 2007; Oliveira et al. 2010). At low dose levels (1 mg/kg-day) permanent changes in glucose homeostasis were observed only when exposure occurred during both fetal and neonatal life (i.e., pregnancy and lactation); however, when dams were exposed to 3 mg/kg-day (Somm et al. 2008) or 6 mg/kg-day (Oliveira et al. 2010), then either fetal or lactational exposure was sufficient to affect glycemic control. Importantly, no effects on glucose homeostasis were observed in Wistar rats (Jose et al. 2009) or in a series of studies (Jose et al. 2009; Swislocki 2003; Swislocki and Fakiri 2008; Swislocki et al. 1997) in Sprague-Dawley rats treated with nicotine after weaning. Taken together, these data suggest that cigarette smoke or nicotine exposure during gestation and lactation is the critical window of exposure.

**Figure 6:**
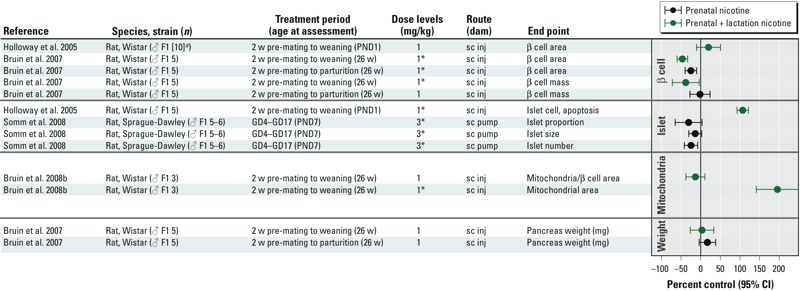
Animal studies of prenatal or prenatal + lactational exposure to nicotine and pancreatic end points. The primary grouping of studies is based on the type of pancreatic effect. Within the effect category, main findings were sorted based on whether treatment occurred only during prenatally (black) or prenatal + lactational (green). Abbreviations: GD, gestational day; PND, postnatal day; sc, subcutaneous; w, weeks. ***^a^***Value was assumed or estimated based on data presented in publication. *Statistically significant effect at specified dose level as reported in publication.

*Pancreatic effects.* Changes in pancreatic weight, morphology, and function have been reported in animals that were treated with nicotine during gestation alone or during gestation and lactation (Bruin et al. 2007, 2008a, 2008b; Grove et al. 2001; Holloway et al. 2005; Somm et al. 2008) (Figure 6). These pancreatic effects include increased β-cell apoptosis and decreased β-cell mass in rodents (Bruin et al. 2007; Holloway et al. 2005; Somm et al. 2009). Other findings include decreased pancreatic weight in infant rhesus monkeys (Grove et al. 2001). As noted earlier, the treatment protocols used in the rodent studies result in maternal serum cotinine levels that are considered relevant to women who smoke or use nicotine patches as cigarette substitutes. The pancreatic effects are hypothesized to occur as a direct effect of nicotine binding to nicotinic acetylcholine receptors in the developing pancreas, leading to oxidative stress and mitochondrial damage and eventual β-cell apoptosis (Bruin et al. 2007, 2008a, 2008b, 2008c). Pancreatic tissue is considered to be especially susceptible to oxidative stress–mediated tissue damage due to low levels of antioxidant enzyme expression (Lenzen et al. 1996; Tiedge et al. 1997).

*Leptin signaling*. Adipose tissue is increasingly recognized as an active endocrine organ with many secretory products. In addition, adipose tissue is considered to be part of the innate immune system (Gaspari et al. 2011). Leptin is one of the key hormones involved in feeding behavior and energy expenditure [reviewed by Belgardt et al. (2010)]. In general terms, leptin is secreted from the adipocytes of white adipose tissue and delivers information on peripheral energy stores to the central nervous system. Leptin resistance that involves impairment in leptin transport, leptin signaling, and/or the neurocircuitry of energy balance is a risk factor for obesity (Morris and Rui 2009). Several studies have assessed leptin levels or the expression of proteins involved in leptin signaling after developmental nicotine exposure (Grove et al. 2001; Huang and Winzer-Serhan 2007; Santos-Silva et al. 2010). Findings from animal studies reveal that early exposure to nicotine appears to be associated with dysregulation of leptin levels, likely via hypertrophy of adipocytes and increased gene expression of pro-adipogenic transcription factors (Somm et al. 2008) and hypothalamic leptin signaling (Oliveira et al. 2009, 2010).

The association between smoking and leptin levels in human infants remains controversial, with both hyper- and hypoleptinemia being reported (Coutant et al. 2001; Helland et al. 2001; Kayemba-Kay’s et al. 2008; Mantzoros et al. 1997; Ozkan et al. 2005; Pardo et al. 2004, 2005; Vatten et al. 2002).

## Conclusions and Research Recommendations

Based on human epidemiological studies and animal experiments reported in the literature, current evidence supports a causal relationship between maternal smoking and increased risk of obesity or overweight in offspring; however, the possibility that the association is attributable to unmeasured residual confounding cannot be completely ruled out. The literature is not sufficient to evaluate the potential impact of maternal smoking after delivery, or the contribution of exposure to secondhand smoke during pregnancy and early childhood. Existing studies in humans do not provide strong support for an association between maternal or parental smoking and childhood T1D, and too few epidemiological studies have assessed outcomes related to T2D to reach conclusions. However, findings from animal studies identify associations between perinatal nicotine exposure and disruption of pathways important in the pathophysiology of T2D, including reduced β-cell mass and impaired β-cell function.

In Appendix 1 we identify research gaps and provide specific study recommendations noting in particular the need to better understand the *a*) relationship between developmental exposure to tobacco smoke as a risk factor for T2D or metabolic syndrome, *b*) consequences of pre- and postnatal exposure to secondhand smoke (also called environmental tobacco smoke, involuntary smoke, and passive smoke), *c*) impact of timing of exposure, dose, duration and route (e.g., nicotine replacement therapy), and *d*) mechanistic basis for the association between cigarette smoke or nicotine exposure with childhood obesity and metabolic outcomes. Elucidating the mechanistic basis for these associations could be an important component of developing a toxicological screening approach that could be used to identify other environmental chemicals for targeted assessment of obesogenic and metabolic effects. For example, a number of pesticides included in Phase 1 of the U.S. Environmental Protection Agency (U.S. EPA 2012) ToxCast™ high throughput screening program interact with nicotinic receptors but have never been assessed for potential adipogenic or metabolic effects [see Supplemental Material, Figure S2 (http://dx.doi.org/10.1289/ehp.1205404)]. Addressing the data gaps can help us understand more about the nature of the association as well as inform decisions regarding intervention strategies. Epidemiological studies will also need to consider the challenges related to validity of self-reported maternal tobacco use or secondhand smoke exposure, especially in studies evaluating the long-term health consequences for offspring where exposure assessment may occur many years after the exposure event. With respect to mechanistic research, more attention should be given to the possibility that environmental exposures could alter central nervous system regulation of energy and body weight homeostasis. The brain is the primary organ involved with determining behavior and obesity; metabolic diseases are often considered to be manifestations of behavioral excess (i.e., consuming calories in excess of one’s energy needs). A number of pathways are important for the development of obesity and/or diabetes, including but not limited to alterations in neurohumoral signaling, feeding behavior, energy balance, brain and peripheral inflammation, and insulin resistance. To date, the effects of maternal cigarette smoking and/or nicotine exposure on these pathways remains largely unexplored (Wilborn et al. 2005; Woods 2009; Woods and D’Alessio 2008; Zheng et al. 2009). Another largely unexplored hypothesis is that fetal/neonatal exposure to smoking/nicotine sensitizes the offspring to adverse effects of obesogenic diets. Although perturbations in the microbiome profile have been associated with diabetes and obesity, the contribution of or interactions with smoking have not been explored and may also influence susceptibility to adverse diets.

The association between maternal smoking and childhood obesity adds to the recognized health burden from tobacco exposure, which is estimated at almost 5 million deaths per year globally (World Health Organization 2010). Maternal smoking during pregnancy may not account for as many cases of childhood obesity or overweight as more traditional risk factors such as decreased physical activity, watching television > 1 hr/day, and low parental education level (Toschke et al. 2007b), but it is a risk factor that, if prevented, can help reduce the burden of childhood obesity at the population level. From a toxicological perspective, the linkages between maternal smoking during pregnancy and childhood overweight/obesity provide proof-of-concept of how early-life exposure to an environmental toxicant can act as a risk factor for childhood obesity.

## Appendix 1. Data Gaps and Research Needs

Epidemiology

- Additional assessment of association between maternal smoking and risk of T2D and metabolic syndrome in offspring
- Clarification of the pre- and postnatal consequences of secondhand smoke on childhood obesity and metabolic outcomes
- Characterization of factors such as timing of exposure (including adolescence), dose, duration and route (e.g., nicotine replacement therapy)
- Evaluate potential confounding by or interactions with postnatal diet and activity levels
- Consider studies of nicotine replacement therapy (NRT) to better establish the association between nicotine and obesity or diabetes and other metabolic disorders in humans

––RT during pregnancy trials, NRT following smoking cessation, nicotine therapeutics for nonsmokers, Snus (a moist powder tobacco product similar to dry snuff)

––Other data may be available from FDA for nicotinic acetylcholine receptor agonist drugs, and putative “reduced” harm cigarettes

Animal and in vitro

- Better understanding of the basic biology of critical cells (i.e., adipocytes, β cells) and their function in health and disease, including how the biology that controls body weight and metabolic set points change with life stage
- Further characterize impact of factors such as timing of exposure, dose, duration, and route (e.g., NRT) on adiposity, diabetes, and metabolic-related health outcomes
- Mechanistic effects of smoking on genomic/epigenomic and molecular targets that coordinate central and peripheral nutrient homeostasis and metabolic function
- Determine the relative contribution of other constituents of cigarette smoke
  ––Graded step wise manner—starting with high throughput–type screening with relevant cell types, then in vivo alone, and then in vivo in combination with nicotine
- Evaluate interactions between fetal/neonatal exposure to smoking/nicotine and postnatal diet and activity levels
- Assess the effects of other chemicals that act as agonists for nicotinic acetylcholine receptors (nAChRs), such as neonicotinoid pesticides as well as chemicals identified in the U.S. EPA’s ToxCast™ high throughput screening program [see Supplemental Material Figure 2 (http://dx.doi.org/10.1289/ehp.1205404)].

## Supplemental Material

(1.1 MB) PDF

## References

[r1] Adams AK, Harvey HE, Prince RJ (2005). Association of maternal smoking with overweight at age 3 y in American Indian children.. Am J Clin Nutr.

[r2] Al Mamun A, Lawlor DA, Alati R, O’Callaghan MJ, Williams GM, Najman JM (2006). Does maternal smoking during pregnancy have a direct effect on future offspring obesity? Evidence from a prospective birth cohort study.. Am J Epidemiol.

[r3] Belgardt BF, Bruning JC (2010). CNS leptin and insulin action in the control of energy homeostasis.. Ann NY Acad Sci.

[r4] Bergmann KE, Bergmann RL, Von Kries R, Bohm O, Richter R, Dudenhausen JW (2003). Early determinants of childhood overweight and adiposity in a birth cohort study: role of breast-feeding.. Int J Obes Relat Metab Disord.

[r5] Beyerlein A Toschke AM Schaffrath Rosario A von Kries R 2011 Risk factors for obesity: further evidence for stronger effects on overweight children and adolescents compared to normal-weight subjects. PLoS One 6 1 e15739; doi:10.1371/journal.pone.0015739 [Online 20 January 2011] 21283747 PMC3024393

[r6] Beyerlein A, Toschke AM, von Kries R (2010). Risk factors for childhood overweight: shift of the mean body mass index and shift of the upper percentiles: results from a cross-sectional study.. Int J Obes (Lond).

[r7] Boerschmann H, Pfluger M, Henneberger L, Ziegler AG, Hummel S (2010). Prevalence and predictors of overweight and insulin resistance in offspring of mothers with gestational diabetes mellitus.. Diabetes Care.

[r8] Braun JM, Daniels JL, Poole C, Olshan AF, Hornung R, Bernert JT (2010). Prenatal environmental tobacco smoke exposure and early childhood body mass index.. Paediatr Perinat Epidemiol.

[r9] Bruin JE, Gerstein HC, Morrison KM, Holloway AC (2008a). Increased pancreatic beta-cell apoptosis following fetal and neonatal exposure to nicotine is mediated via the mitochondria.. Toxicol Sci.

[r10] Bruin JE, Kellenberger LD, Gerstein HC, Morrison KM, Holloway AC (2007). Fetal and neonatal nicotine exposure and postnatal glucose homeostasis: identifying critical windows of exposure.. J Endocrinol.

[r11] Bruin JE, Petre MA, Lehman MA, Raha S, Gerstein HC, Morrison KM (2008b). Maternal nicotine exposure increases oxidative stress in the offspring.. Free Radic Biol Med.

[r12] Bruin JE Petre MA Raha S Morrison KM Gerstein HC Holloway AC 2008c Fetal and neonatal nicotine exposure in Wistar rats causes progressive pancreatic mitochondrial damage and beta cell dysfunction. PLoS One 3 10 e3371; doi:10.1371/journal.pone.0003371 [Online 8 October 2008] 18852877 PMC2566598

[r13] CDC (Centers for Disease Control and Prevention) (2011). Diabetes Data and Trends.. http://apps.nccd.cdc.gov/DDTSTRS/default.aspx.

[r14] Chen A, Pennell ML, Klebanoff MA, Rogan WJ, Longnecker MP (2006). Maternal smoking during pregnancy in relation to child overweight: follow-up to age 8 years.. Int J Epidemiol.

[r15] Chen H Iglesias MA Caruso V Morris MJ 2011 Maternal cigarette smoke exposure contributes to glucose intolerance and decreased brain insulin action in mice offspring independent of maternal diet. PloS one 6 11 e27260; doi:10.1371/journal.pone.0027260 [Online 4 November 2011] 22076142 PMC3208635

[r16] Christou H, Connors JM, Ziotopoulou M, Hatzidakis V, Papathanassoglou E, Ringer SA (2001). Cord blood leptin and insulin-like growth factor levels are independent predictors of fetal growth.. J Clin Endocrinol Metab.

[r17] Coutant R, Boux de Casson F, Douay O, Mathieu E, Rouleau S, Beringue F (2001). Relationships between placental GH concentration and maternal smoking, newborn gender, and maternal leptin: possible implications for birth weight.. J Clin Endocrinol Metab.

[r18] Cupul-Uicab LA, Skjaerven R, Haug K, Melve KK, Engel SM, Longnecker MP (2011). *In utero* exposure to maternal tobacco smoke and subsequent obesity, hypertension, and gestational diabetes among women in the MoBa cohort.. Environ Health Perspect.

[r19] Dahlquist G, Kallen B. (1992). Maternal-child blood group incompatibility and other perinatal events increase the risk for early-onset type 1 (insulin-dependent) diabetes mellitus.. Diabetologia.

[r20] Dahlquist GG, Nystrom L, Patterson CC (2011). Incidence of type 1 diabetes in Sweden among individuals aged 0–34 years, 1983–2007: an analysis of time trends.. Diabetes Care.

[r21] DIAMOND Project Group (2006). Incidence and trends of childhood Type 1 diabetes worldwide 1990–1999.. Diabet Med.

[r22] Dubois L, Girard M. (2006). Early determinants of overweight at 4.5 years in a population-based longitudinal study.. Int J Obes (Lond).

[r23] Durmus B, Ay L, Hokken-Koelega AC, Raat H, Hofman A, Steegers EA (2011a). Maternal smoking during pregnancy and subcutaneous fat mass in early childhood. The Generation R Study.. Eur J Epidemiol.

[r24] Durmus B, Kruithof CJ, Gillman MH, Willemsen SP, Hofman A, Raat H (2011b). Parental smoking during pregnancy, early growth, and risk of obesity in preschool children: the Generation R Study.. Am J Clin Nutr.

[r25] Edwards JR, Prozialeck WC (2009). Cadmium, diabetes and chronic kidney disease.. Toxicol Appl Pharmacol.

[r26] Eyre H, Kahn R, Robertson RM (2004). Preventing cancer, cardiovascular disease, and diabetes: a common agenda for the American Cancer Society, the American Diabetes Association, and the American Heart Association.. CA Cancer J Clin.

[r27] Fasting MH, Oien T, Storro O, Nilsen TI, Johnsen R, Vik T (2009). Maternal smoking cessation in early pregnancy and offspring weight status at four years of age. A prospective birth cohort study.. Early Hum Devel.

[r28] Freedman DS, Sherry B (2009). The validity of BMI as an indicator of body fatness and risk among children.. Pediatrics.

[r29] Gao YJ, Holloway AC, Su LY, Takemori K, Lu C, Lee RM (2008). Effects of fetal and neonatal exposure to nicotine on blood pressure and perivascular adipose tissue function in adult life.. Eur J Pharmacol.

[r30] Gao YJ, Holloway AC, Zeng ZH, Lim GE, Petrik JJ, Foster WG (2005). Prenatal exposure to nicotine causes postnatal obesity and altered perivascular adipose tissue function.. Obes Res.

[r31] Gaspari L, Paris F, Jandel C, Kalfa N, Orsini M, Daures JP (2011). Prenatal environmental risk factors for genital malformations in a population of 1442 French male newborns: a nested case-control study.. Hum Reprod.

[r32] Gillman MW, Rifas-Shiman SL, Kleinman K, Oken E, Rich-Edwards JW, Taveras EM (2008). Developmental origins of childhood overweight: potential public health impact.. Obesity.

[r33] Gilman SE, Gardener H, Buka SL (2008). Maternal smoking during pregnancy and children’s cognitive and physical development: a causal risk factor?. Am J Epidemiol.

[r34] Goldani MZ, Haeffner LS, Agranonik M, Barbieri MA, Bettiol H, Silva AA (2007). Do early life factors influence body mass index in adolescents?. Braz J Med Biol Res.

[r35] Gorog K, Pattenden S, Antova T, Niciu E, Rudnai P, Scholtens S (2009). Maternal smoking during pregnancy and childhood obesity: results from the CESAR study.. Matern Child Health J.

[r36] Griffiths LJ, Hawkins SS, Cole TJ, Dezateux C (2010). Risk factors for rapid weight gain in preschool children: findings from a UK-wide prospective study.. Int J Obes (Lond).

[r37] Grove KL, Sekhon HS, Brogan RS, Keller JA, Smith MS, Spindel ER (2001). Chronic maternal nicotine exposure alters neuronal systems in the arcuate nucleus that regulate feeding behavior in the newborn rhesus macaque.. J Clin Endocrinol Metab.

[r38] Hackman R, Kapur B, Koren G. (1999). Use of the nicotine patch by pregnant women. New Engl J Med.

[r39] Hathout EH, Beeson WL, Ischander M, Rao R, Mace JW (2006). Air pollution and type 1 diabetes in children.. Pediatr Diabetes.

[r40] Helland IB, Reseland JE, Saugstad OD, Drevon CA (2001). Smoking related to plasma leptin concentration in pregnant women and their newborn infants.. Acta Paediatr.

[r41] Hill SY, Shen S, Locke Wellman J, Rickin E, Lowers L (2005). Offspring from families at high risk for alcohol dependence: increased body mass index in association with prenatal exposure to cigarettes but not alcohol.. Psychiatry Res.

[r42] Holloway AC, Cuu DQ, Morrison KM, Gerstein HC, Tarnopolsky MA (2007). Transgenerational effects of fetal and neonatal exposure to nicotine.. Endocrine.

[r43] Holloway AC, Lim GE, Petrik JJ, Foster WG, Morrison KM, Gerstein HC (2005). Fetal and neonatal exposure to nicotine in Wistar rats results in increased beta cell apoptosis at birth and postnatal endocrine and metabolic changes associated with type 2 diabetes.. Diabetologia.

[r44] Horta BL, Gigante DP, Nazmi A, Silveira VM, Oliveira I, Victora CG (2011). Maternal smoking during pregnancy and risk factors for cardiovascular disease in adulthood.. Atherosclerosis.

[r45] Huang LZ, Winzer-Serhan UH (2007). Nicotine regulates mRNA expression of feeding peptides in the arcuate nucleus in neonatal rat pups.. Dev Neurobiol.

[r46] Huang RC, Burke V, Newnham JP, Stanley FJ, Kendall GE, Landau LI (2007). Perinatal and childhood origins of cardiovascular disease.. Int J Obes (Lond).

[r47] Hummel M, Schenker M, Ziegler AG (2001). Influence of perinatal factors on the appearance of islet autoantibodies in offspring of parents with type 1 diabetes.. Pediatr Diabetes.

[r48] Iliadou AN, Koupil I, Villamor E, Altman D, Hultman C, Langstrom N (2010). Familial factors confound the association between maternal smoking during pregnancy and young adult offspring overweight.. Int J Epidemiol.

[r49] Ino T. (2010). Maternal smoking during pregnancy and offspring obesity: meta-analysis.. Pediatr Int.

[r50] Ino T, Shibuya T, Saito K, Ohtani T. (2011). Effects of maternal smoking during pregnancy on body composition in offspring.. Pediatr Int.

[r51] Johansson A, Hermansson G, Ludvigsson J. (2008). Tobacco exposure and diabetes-related autoantibodies in children: results from the ABIS study.. Ann NY Acad Sci.

[r52] Jose AS, Franciscato C, Sonego F, Figueiro M, Thiesen FV, Garcia SC (2009). Sensitivity of young rats to nicotine exposure: Physiological and biochemical parameters.. Ecotoxicol Environ Saf.

[r53] Kanellopoulos TA, Varvarigou AA, Karatza AA, Beratis NG (2007). Course of growth during the first 6 years in children exposed *in utero* to tobacco smoke.. Eur J Pediatr.

[r54] Karaolis-Danckert N, Buyken AE, Kulig M, Kroke A, Forster J, Kamin W (2008). How pre- and postnatal risk factors modify the effect of rapid weight gain in infancy and early childhood on subsequent fat mass development: results from the Multicenter Allergy Study 90.. Am J Clin Nutr.

[r55] Kayemba-Kay’s S, Geary MP, Pringle J, Rodeck CH, Kingdom JC, Hindmarsh PC. 2008 Gender, smoking during pregnancy and gestational age influence cord leptin concentrations in newborn infants. Eur J Endocrinol 159 3 217 224 18524794 10.1530/EJE-08-0171 PMC2754114

[r56] Kim J, Peterson KE, Scanlon KS, Fitzmaurice GM, Must A, Oken E (2006). Trends in overweight from 1980 through 2001 among preschool-aged children enrolled in a health maintenance organization.. Obesity (Silver Spring).

[r57] Koshy G, Delpisheh A, Brabin BJ (2011). Dose response association of pregnancy cigarette smoke exposure, childhood stature, overweight and obesity.. Eur J Public Health.

[r58] Koupil I, Toivanen P. (2008). Social and early-life determinants of overweight and obesity in 18-year-old Swedish men.. Int J Obes (Lond).

[r59] Kuhle S, Allen AC, Veugelers PJ (2010). Perinatal and childhood risk factors for overweight in a provincial sample of Canadian Grade 5 students.. Int J Pediatr Obes.

[r60] Kwok MK, Schooling CM, Lam TH, Leung GM (2010). Paternal smoking and childhood overweight: evidence from the Hong Kong “Children of 1997.”. Pediatrics.

[r61] Leary SD, Smith GD, Rogers IS, Reilly JJ, Wells JC, Ness AR (2006). Smoking during pregnancy and offspring fat and lean mass in childhood.. Obesity (Silver Spring).

[r62] Lee MJ, Fried SK (2009). Integration of hormonal and nutrient signals that regulate leptin synthesis and secretion.. Am J Physiol Endocrinol Metab.

[r63] Lenzen S, Drinkgern J, Tiedge M. (1996). Low antioxidant enzyme gene expression in pancreatic islets compared with various other mouse tissues.. Free Radic Biol Med.

[r64] Lumley J Chamberlain C Dowswell T Oliver S Oakley L Watson L. 2008 Interventions for promoting smoking cessation during pregnancy. Cochrane Database Syst Rev 18:(4 CD001055; DOI: [Online 27 July 1998; last assessed as up to date, 4 December 2008] 10.1002/14651858.CD001055.pub3 PMC4090746 19588322

[r65] Mantzoros CS, Varvarigou A, Kaklamani VG, Beratis NG, Flier JS (1997). Effect of birth weight and maternal smoking on cord blood leptin concentrations of full-term and preterm newborns.. J Clin Endocrinol Metab.

[r66] Marshall AL, Chetwynd A, Morris JA, Placzek M, Smith C, Olabi A (2004). Type 1 diabetes mellitus in childhood: a matched case control study in Lancashire and Cumbria, UK.. Diabet Med.

[r67] Matijasevich A, Brion MJ, Menezes AM, Barros AJ, Santos IS, Barros FC (2011). Maternal smoking during pregnancy and offspring growth in childhood: 1993 and 2004 Pelotas cohort studies.. Arch Dis Child.

[r68] Mendez MA, Torrent M, Ferrer C, Ribas-Fito N, Sunyer J (2008). Maternal smoking very early in pregnancy is related to child overweight at age 5–7 y.. Am J Clin Nutr.

[r69] Mizutani T, Suzuki K, Kondo N, Yamagata Z. (2007). Association of maternal lifestyles including smoking during pregnancy with childhood obesity.. Obesity (Silver Spring).

[r70] Montgomery SM, Ekbom A (2002). Smoking during pregnancy and diabetes mellitus in a British longitudinal birth cohort.. BMJ.

[r71] Morris DL, Rui L (2009). Recent advances in understanding leptin signaling and leptin resistance.. Am J Physiol Endocrinol Metab.

[r72] National Toxicology Program (2011). NTP Workshop: Role of Environmental Chemicals in the Development of Diabetes and Obesity.. http://ntp.niehs.nih.gov/?objectid=49E4B077-C108-8BBA-25B2F05DE614C9C4.

[r73] Newman MB, Shytle RD, Sanberg PR (1999). Locomotor behavioral effects of prenatal and postnatal nicotine exposure in rat offspring.. Behav Pharmacol.

[r74] Ng SP, Conklin DJ, Bhatnagar A, Bolanowski DD, Lyon J, Zelikoff JT (2009). Prenatal exposure to cigarette smoke induces diet- and sex-dependent dyslipidemia and weight gain in adult murine offspring.. Environ Health Perspect.

[r75] NIDDK (National Institute of Diabetes and Digestive and Kidney Diseases) (2011). Diabetes Research Strategic Plan.. http://www2.niddk.nih.gov/AboutNIDDK/ReportsAndStrategicPlanning/DiabetesPlan/PlanPosting.htm.

[r76] NIH (National Institutes of Health) Obesity Research Task Force (2011). Strategic Plan for NIH Obesity Research.. http://www.obesityresearch.nih.gov/about/strategic-plan.aspx.

[r77] Ogden C, Carroll M (2010). Prevalence of Obesity Among Children and Adolescents: United States, Trends 1963–1965 Through 2007–2008. CDC-NCHS Health E-Stat.. http://www.cdc.gov/nchs/data/hestat/obesity_child_07_08/obesity_child_07_08.htm.

[r78] Oken E, Huh SY, Taveras EM, Rich-Edwards JW, Gillman MW (2005). Associations of maternal prenatal smoking with child adiposity and blood pressure.. Obes Res.

[r79] Oken E, Levitan EB, Gillman MW (2008). Maternal smoking during pregnancy and child overweight: systematic review and meta-analysis.. Int J Obes (Lond).

[r80] Oliveira E, Moura EG, Santos-Silva AP, Fagundes AT, Rios AS, Abreu-Villaca Y (2009). Short- and long-term effects of maternal nicotine exposure during lactation on body adiposity, lipid profile, and thyroid function of rat offspring.. J Endocrinol.

[r81] Oliveira E, Moura EG, Santos-Silva AP, Pinheiro CR, Lima N, Nogueira-Neto J (2010). Neonatal nicotine exposure causes insulin and leptin resistance and inhibits hypothalamic leptin signaling in adult rat offspring.. J Endocrinol.

[r82] Ozkan B, Ermis B, Tastekin A, Doneray H, Yildirim A, Ors R. (2005). Effect of smoking on neonatal and maternal serum and breast milk leptin levels.. Endocr Res.

[r83] Pardo IM, Geloneze B, Tambascia MA, Barros AA (2005). Inverse relationship between cord blood adiponectin concentrations and the number of cigarettes smoked during pregnancy.. Diabetes Obes Metab.

[r84] Pardo IM, Geloneze B, Tambascia MA, Barros-Filho AA (2004). Does maternal smoking influence leptin levels in term, appropriate-for-gestational-age newborns?. J Matern Fetal Neonatal Med.

[r85] Patterson CC, Dahlquist GG, Gyurus E, Green A, Soltész G, EURODIAB Study Group (2009). Incidence trends for childhood type 1 diabetes in Europe during 1989–2003 and predicted new cases 2005–20: a multicentre prospective registration study.. Lancet.

[r86] Pelleymounter MA, Cullen MJ, Baker MB, Hecht R, Winters D, Boone T (1995). Effects of the obese gene product on body weight regulation in ob/ob mice.. Science.

[r87] Power C, Atherton K, Thomas C. (2010). Maternal smoking in pregnancy, adult adiposity and other risk factors for cardiovascular disease.. Atherosclerosis.

[r88] Power C, Jefferis BJ (2002). Fetal environment and subsequent obesity: a study of maternal smoking.. Int J Epidemiol.

[r89] Prescott SL (2008). Effects of early cigarette smoke exposure on early immune development and respiratory disease.. Paediatr Respir Rev.

[r90] Rabinoff M, Caskey N, Rissling A, Park C. (2007). Pharmacological and chemical effects of cigarette additives.. Am J Public Health.

[r91] Raum E, Kupper-Nybelen J, Lamerz A, Hebebrand J, Herpertz-Dahlmann B, Brenner H. (2011). Tobacco smoke exposure before, during, and after pregnancy and risk of overweight at age 6.. Obesity (Silver Spring, Md).

[r92] Ravnborg TL, Jensen TK, Andersson AM, Toppari J, Skakkebaek NE, Jorgensen N (2011). Prenatal and adult exposures to smoking are associated with adverse effects on reproductive hormones, semen quality, final height and body mass index.. Hum Reprod.

[r93] Reilly JJ, Armstrong J, Dorosty AR, Emmett PM, Ness A, Rogers I (2005). Early life risk factors for obesity in childhood: Cohort study.. BMJ.

[r94] Reilly JJ Kelly J 2011. Long-term impact of overweight and obesity in childhood and adolescence on morbidity and premature mortality in adulthood: systematic review. Int J Obes (2005) 35 7 891 898 10.1038/ijo.2010.222 20975725

[r95] Robertson L Harrild K. 2010 Maternal and neonatal risk factors for childhood type 1 diabetes: a matched case-control study. BMC Public Health 10 281; doi:10.1186/1471-2458-10-281 [Online 27 May 2010] 20507546 PMC2885337

[r96] Rooney BL, Mathiason MA, Schauberger CW (2010). Predictors of obesity in childhood, adolescence, and adulthood in a birth cohort.. Matern Child Health J.

[r97] Rosenbauer J, Herzig P, Kaiser P, Giani G. (2007). Early nutrition and risk of type 1 diabetes mellitus—a nationwide case-control study in preschool children.. Exp Clin Endocrinol Diabetes.

[r98] Salsberry PJ, Reagan PB (2005). Dynamics of early childhood overweight.. Pediatrics.

[r99] Salsberry PJ, Reagan PB (2007). Taking the long view: the prenatal environment and early adolescent overweight.. Res Nurs Health.

[r100] Samper MP, Jimenez-Muro A, Nerin I, Marqueta A, Ventura P, Rodriguez G (2011). Maternal active smoking and newborn body composition.. Early Hum Dev.

[r101] Santos-Silva AP, Moura EG, Pinheiro CR, Rios AS, Abreu-Villaca Y, Passos MC (2010). Neonatal nicotine exposure alters leptin signaling in the hypothalamus-pituitary-thyroid axis in the late postnatal period and adulthood in rats.. Life Sci.

[r102] Santos-Silva AP, Oliveira E, Pinheiro CR, Nunes-Freitas AL, Abreu-Villaca Y, Santana AC (2011). Effects of tobacco smoke exposure during lactation on nutritional and hormonal profiles in mothers and offspring.. J Endocrinol.

[r103] Sharma AJ, Cogswell ME, Li R (2008). Dose-response associations between maternal smoking during pregnancy and subsequent childhood obesity: effect modification by maternal race/ethnicity in a low-income US cohort.. Am J Epidemiol.

[r104] Silva AA, Vasconcelos AG, Bettiol H, Barbieri MA (2010). Socioeconomic status, birth weight, maternal smoking during pregnancy and adiposity in early adult life: an analysis using structural equation modeling.. Cad Saude Publica.

[r105] Sipetic SB, Vlajinac HD, Kocev NI, Marinkovic JM, Radmanovic SZ, Bjekic MD (2005). The Belgrade childhood diabetes study: a multivariate analysis of risk determinants for diabetes.. Eur J Public Health.

[r106] Skrodeniene E, Marciulionyte D, Padaiga Z, Jasinskiene E, Sadauskaite-Kuehne V, Ludvigsson J. (2008). Environmental risk factors in prediction of childhood prediabetes.. Medicina (Kaunas).

[r107] Somm E, Schwitzgebel VM, Vauthay DM, Aubert ML, Huppi PS (2009). Prenatal nicotine exposure and the programming of metabolic and cardiovascular disorders.. Mol Cell Endocrinol.

[r108] Somm E, Schwitzgebel VM, Vauthay DM, Camm EJ, Chen CY, Giacobino JP (2008). Prenatal nicotine exposure alters early pancreatic islet and adipose tissue development with consequences on the control of body weight and glucose metabolism later in life.. Endocrinology.

[r109] Stene LC, Barriga K, Norris JM, Hoffman M, Erlich HA, Eisenbarth GS (2004). Perinatal factors and development of islet autoimmunity in early childhood: the diabetes autoimmunity study in the young.. Am J Epidemiol.

[r110] Steur M, Smit HA, Schipper CM, Scholtens S, Kerkhof M, de Jongste JC, et al 2010 Predicting the risk of newborn children to become overweight later in childhood: The PIAMA birth cohort study. Int J Pediatr Obes 6(2-2):e170–e178. 10.3109/17477166.2010.519389 20883125

[r111] Suzuki K, Ando D, Sato M, Tanaka T, Kondo N, Yamagata Z. (2009). The association between maternal smoking during pregnancy and childhood obesity persists to the age of 9–10 years.. J Epidemiol.

[r112] Suzuki K, Kondo N, Sato M, Tanaka T, Ando D, Yamagata Z. (2011). Gender differences in the association between maternal smoking during pregnancy and childhood growth trajectories: multilevel analysis.. Int J Obes (Lond).

[r113] Svensson J, Carstensen B, Mortensen HB, Borch-Johnsen K (2005). Early childhood risk factors associated with type 1 diabetes—is gender important?. Eur J Epidemiol.

[r114] Swislocki AL (2003). Smokeless nicotine administration does not result in hypertension or a deterioration in glucose tolerance or insulin sensitivity in juvenile rats.. Metabolism.

[r115] Swislocki AL, Fakiri Z (2008). Smokeless nicotine exposure has no lasting effect on fasting or postglucose circulation leptin in young rats.. Metab Syndr Relat Disord.

[r116] Swislocki AL, Tsuzuki A, Tait M, Khuu D, Fann K (1997). Smokeless nicotine administration is associated with hypertension but not with a deterioration in glucose tolerance in rats.. Metabolism.

[r117] Syme C, Abrahamowicz M, Mahboubi A, Leonard GT, Perron M, Richer L (2010). Prenatal exposure to maternal cigarette smoking and accumulation of intra-abdominal fat during adolescence.. Obesity (Silver Spring).

[r118] Thiering E, Bruske I, Kratzsch J, Thiery J, Sausenthaler S, Meisinger C (2011). Prenatal and postnatal tobacco smoke exposure and development of insulin resistance in 10 year old children.. Int J Hyg Environ Health.

[r119] Thomas C, Hypponen E, Power C. (2007). Prenatal exposures and glucose metabolism in adulthood: are effects mediated through birth weight and adiposity?. Diabetes Care.

[r120] Tiedge M, Lortz S, Drinkgern J, Lenzen S. (1997). Relation between antioxidant enzyme gene expression and antioxidative defense status of insulin-producing cells.. Diabetes.

[r121] Tome FS, Cardoso VC, Barbieri MA, Silva AA, Simoes VM, Garcia CA (2007). Are birth weight and maternal smoking during pregnancy associated with malnutrition and excess weight among school age children?. Braz J Med Biol Res.

[r122] Toschke AM, Ehlin A, Koletzko B, Montgomery SM (2007a). Paternal smoking is associated with a decreased prevalence of type 1 diabetes mellitus among offspring in two national British birth cohort studies (NCDS and BCS70).. J Perinat Med.

[r123] Toschke AM, Koletzko B, Slikker W, Hermann M, von Kries R (2002). Childhood obesity is associated with maternal smoking in pregnancy.. Eur J Pediatr.

[r124] Toschke AM, Montgomery SM, Pfeiffer U, von Kries R (2003). Early intrauterine exposure to tobacco-inhaled products and obesity.. Am J Epidemiol.

[r125] Toschke AM, Ruckinger S, Bohler E, Von Kries R (2007b). Adjusted population attributable fractions and preventable potential of risk factors for childhood obesity.. Public Health Nutr.

[r126] U.S. EPA (U.S. Environmental Protection Agency) (2012). ToxCast™.. http://actor.epa.gov/actor/faces/ToxCastDB/Home.jsp.

[r127] Vatten LJ, Nilsen ST, Odegard RA, Romundstad PR, Austgulen R (2002). Insulin-like growth factor I and leptin in umbilical cord plasma and infant birth size at term.. Pediatrics.

[r128] Vik T, Jacobsen G, Vatten L, Bakketeig LS (1996). Pre- and post-natal growth in children of women who smoked in pregnancy.. Early Hum Dev.

[r129] von Kries R, Bolte G, Baghi L, Toschke AM, GMESG (2008). Parental smoking and childhood obesity—is maternal smoking in pregnancy the critical exposure?. Int J Epidemiol.

[r130] von Kries R, Toschke AM, Koletzko B, Slikker W (2002). Maternal smoking during pregnancy and childhood obesity.. Am J Epidemiol.

[r131] von Schnurbein J, Klenk J, Galm C, Berg S, Gottmann P, Steinacker JM (2011). Reference values and early determinants of intra-abdominal fat mass in primary school children.. Horm Res Paediatr.

[r132] Wadsworth EJ, Shield JP, Hunt LP, Baum JD (1997). A case-control study of environmental factors associated with diabetes in the under 5s.. Diabet Med.

[r133] Wahlberg J, Vaarala O, Ludvigsson J. (2011). Asthma and allergic symptoms and type 1 diabetes-related autoantibodies in 2.5-yr-old children.. Pediatr Diabetes.

[r134] Wells JC (2001). A critique of the expression of paediatric body composition data.. Arch Dis Child.

[r135] Wen X, Triche EW, Hogan JW, Shenassa ED, Buka SL (2011). Prenatal factors for childhood blood pressure mediated by intrauterine and/or childhood growth?. Pediatrics.

[r136] Whitaker RC (2004). Predicting preschooler obesity at birth: the role of maternal obesity in early pregnancy.. Pediatrics.

[r137] White House Task Force on Childhood Obesity (2010). Report to the President: Solving the Problem of Childhood Obesity Within a Generation.. http://www.letsmove.gov/sites/letsmove.gov/files/TaskForce_on_Childhood_Obesity_May2010_FullReport.pdf.

[r138] Widerøe M, Torstein V, Jacobsen G, Bakketeig L. (2003). Does maternal smoking during pregnancy cause childhood overweight?. Paediatr Perinat Epidemiol.

[r139] Wilborn C, Beckham J, Campbell B, Harvey T, Galbreath M, La Bounty P (2005). Obesity: prevalence, theories, medical consequences, management, and research directions.. J Int Soc Sports Nutr.

[r140] Williams CM, Kanagasabai T (1984). Maternal adipose tissue response to nicotine administration in the pregnant rat: effects on fetal body fat and cellularity.. Br J Nutr.

[r141] Woods SC (2009). The control of food intake: behavioral versus molecular perspectives.. Cell Metab.

[r142] Woods SC, D’Alessio DA (2008). Central control of body weight and appetite.. J Clin Endocrinol Metab.

[r143] World Health Organization (2010). Global Status Report on Noncommunicable Diseases 2010.. http://www.who.int/nmh/publications/ncd_report_full_en.pdf.

[r144] Zheng H, Lenard NR, Shin AC, Berthoud HR (2009). Appetite control and energy balance regulation in the modern world: reward-driven brain overrides repletion signals.. Int J Obes (Lond).

