# Supplementary material for: Evaluation of the Association between Maternal Smoking, Childhood Obesity, and Metabolic Disorders: A National Toxicology Program Workshop Review
Source: Environ Health Perspect. 2012 Dec 11;121(2):170–80. doi: 10.1289/ehp.1205404 (PMC3569686; doi:10.1289/ehp.1205404)
Supplement: (1.1 MB) PDF [file ehp.1205404.s001.pdf]

SUPPLEMENTAL MATERIAL

Evaluation of the Association Between Maternal Smoking, Childhood Obesity, and Metabolic Disorders: A National Toxicology Program Workshop Review

Mamta Behl, Deepa Rao, Kjersti Aagaard, Terry L. Davidson, Edward D. Levin, Theodore A. Slotkin, Supriya Srinivasan, David Wallinga, Morris F. White, Vickie R. Walker, Kristina A. Thayer, Alison C. Holloway

Table of Contents

Literature search strategy 2

Supplemental Material Figure S1. Study Flow 4

Supplemental Material Table S1. Human studies on maternal smoking during pregnancy and findings related to growth in children 5

Supplemental Material Table S2. Human studies on maternal smoking during pregnancy and findings related to adiposity in children 8

Supplemental Material Figure S2. Nicotinic receptor targets in ToxCast™ 10

References 11

Literature search strategy

MeSH-based PubMed search: (Smoking[mh] OR tobacco smoke pollution[mh]) AND ((maternal exposure[mh] OR “maternal-fetal exchange”[mh] OR maternal behavior[mh] OR pregnancy[mh] OR “prenatal exposure delayed effects”[mh] OR pregnancy outcome[mh] OR fetal development[mh]) OR ((infant[mh] OR “infant, newborn”[mh] OR mothers[mh] OR female[mh] OR “animals, newborn”[mh]) AND (pregnan* OR in utero OR antepartum OR prenat* OR pre-natal OR peripartum OR peri-natal OR perinat* OR intrapartum OR postnat* OR postpartum OR fetal OR fetus OR foetus OR neonat*))) AND (("obesity"[mh] OR "body mass index"[mh] OR "weight gain"[mh] OR "adipogenesis"[mh] OR "adipose tissue"[mh] OR "adipokines"[mh] OR "adiponectin"[mh] OR "leptin"[mh] OR "resistin"[mh]) OR ("diabetes mellitus"[mh] OR "glucose metabolism disorders"[mh] OR "insulin"[mh] OR "insulin resistance"[mh] OR "blood glucose"[mh] OR "islets of langerhans"[mh])) AND (("obesity"[mh] OR "body mass index"[mh] OR "weight gain"[mh] OR "adipogenesis"[mh] OR "adipose tissue"[mh] OR "adipokines"[mh] OR "adiponectin"[mh] OR "leptin"[mh] OR "resistin"[mh]) OR ("diabetes mellitus"[mh] OR "glucose metabolism disorders"[mh] OR "insulin"[mh] OR "insulin resistance"[mh] OR "blood glucose"[mh] OR "islets of langerhans"[mh]))

Keyword-strategy to search "new" un-indexed articles: (smoke* OR smoking) AND (woman OR women OR mother* OR maternal* OR dams) AND (pregnan* OR in utero OR antepartum OR prenat* OR pre-natal OR peripartum OR peri-natal OR perinat* OR intrapartum OR postnat* OR postpartum OR fetal OR fetus OR foetus OR neonat * OR newborn OR baby OR babies OR infant* OR pup OR pups OR offspring) AND (in process[sb] OR publisher[sb]) AND ((diabetes OR "glucose tolerance" OR "glucose intolerance" OR hyperglycemia OR hypoglycemia OR

insulin OR "blood glucose" OR "metabolic syndrome" OR "syndrome x" OR "islets of langerhans") OR (obes* OR "body mass index" OR "body fat" OR "weight gain" OR adipos* OR adipogen* OR adipokine* OR leptin OR resistin OR adiponectin*)) AND (publisher[sb] OR "in process"[sb])

Supplemental Material Figure S1. Study Flow

Based on literature search conducted October 23, 2011

References identified from PubMed search (n = 1,551)

Excluded (n = 1,452)

- (1) No original data on exposure to parental smoking during pregnancy and measures of diabetes, glucose homeostasis, metabolic syndrome, overweight/obesity, or adiposity assessed later than 1 year of age (2) primarily mechanistic or other supportive data (n = 1,378)
- No original data, e.g., review or commentary (n = 14)

↓

Included from PubMed Search (n = 99)
Identified from other sources (n = 2)

<u>Total included (n=101)</u>
Epidemiologic studies (n = 83)
Animal Studies (n = 18)

Supplemental Material Table S1. Human studies on maternal smoking during pregnancy and findings related to growth in children

Reference	Cohort Description	Outcome	Adjustment Factors
(Beyerlein et al. 2010)<br>cross-sectional	Germany[Bavaria] 5-6y, ♂♀<br>N analysis (total N): 9,698	yes vs no, maternal smoking, pregnancy<br>BMI standard deviation score<br>(+) assoc. [adjβ(95%CI): 0.11(0.04,0.18)]	maternal BMI, formula feeding, weight gain first 2 years, low parental education, TV viewing,
(Beyerlein et al. 2011)	Germany[nat'l] KiGGS<br>3-17y, ♂♀<br>N analysis (total N): 11,788(12,383)	yes vs no, maternal smoking, pregnancy<br>BMI standard deviation score<br>(+) assoc. [adjβ(95%CI): 0.31(0.25,0.36)]	maternal BMI, maternal age at birth, TV viewing, migration background, formula-feeding, parental SES
	3-10y, ♂♀<br>N analysis (total N): 7,237	yes vs no, maternal smoking, pregnancy<br>BMI z-score<br>(+) assoc. [adjβ(95%CI): 0.22(0.16,0.28)]	
	11-17y, ♂♀<br>N analysis (total N): 5,986	yes vs no, maternal smoking, pregnancy<br>BMI z-score<br>(+) assoc. [adjβ(95%CI): 0.3(0.22,0.38)]	
(Durmus et al. 2011)<br>prospective	Netherlands[Gen R] 2y, ♂♀<br>N analysis (total N): 671(907)	yes (>5/d) vs no, maternal smoking, pregnancy<br>BMI<br>no assoc. [adjβ(95%CI): 0.02(-0.4,0.45)]	child's age at visit, sex, maternal education, maternal height and weight, breastfeeding ever
(Goldani et al. 2007)<br>prospective	Brazil[Ribeirao Preto] 18y, ♂<br>N analysis (total N): 1,150(6,827)	yes vs no, maternal smoking, pregnancy<br>BMI<br>(+) assoc. [adjβ(95%CI): 0.66(0.18,1.14)]	social class, gestational age, social class at birth, conscript schooling
(Griffiths et al. 2010)<br>prospective	UK[nat'l] Millenium, 3-5y, ♂♀<br>N analysis (total N): 10,920(11,611)	yes vs no, maternal smoking, pregnancy<br>rapid weight gain<br>1.23(95%CI: 1.09,1.38) adjOR	ethnicity, maternal pre-pregnancy overweight, maternal/paternal overweight, # children in household, smoking in same room as child
(Hesketh et al. 2009)<br>prospective	Australia[Victoria] HOYVS, 8-13y, ♂♀<br>N analysis (total N): 1,234(1,569)	yes vs no, maternal smoking, pregnancy<br>BMI, z-score Δ<br>no assoc. [adjβ(95%CI): 0.07(-0.02,0.15)]	baseline BMI, frequency of take away meals, food intake compared to peers, total screen time, siblings, residence, maternal BMI
(Hill et al. 2005)<br>retrospective	US[Pittsburgh, PA] 8-11y, ♂♀<br>N analysis (total N): 288	yes vs no, maternal smoking, pregnancy<br>BMI<br>↑ BMI w/maternal smokinging [$\chi 2=9.94$, $p<0.05$]	familial risk status, prenatal exposure, gender, family ID, maternal MDD diagnosis
(Iliadou et al. 2010)<br>prospective	Sweden[nat'l] milt. serv. registry 17-24y, ♂<br>N analysis (total N): 124203	yes vs no, maternal smoking, pregnancy<br>BMI<br>(+) assoc. [adjβ(95%CI): 0.69(0.62,0.75)]	maternal age, height, BMI and pregnancy weight gain, maternal and paternal socio-economic category and education, offspring urban living, birth weight, head circumference, gestational age and

Supplemental Material Table S1 cont. Human studies on maternal smoking during pregnancy and findings related to growth in children

Reference	Cohort Description	Outcome	Adjustment Factors
			age at conscription
(Kanellopoulos et al. 2007)<br>prospective	Greece[Patras] 6y, ♂♀<br>N analysis (total N): 200(408)	≥15 cig/d vs no, maternal smoking, pregnancy<br>BMI<br>no diff. [normalized effect size,% control(95%CI): 0.6(-4.8,6)]	unadjusted
(Karaolis-Danckert et al. 2008)<br>prospective	Germany[multi-site] MAS-90, 2-6y, ♂♀<br>N analysis (total N): 370	yes vs no, maternal smoking, pregnancy<br>BMI, rate Δ<br>(+) assoc. [adjβ(SE): 0.06(0.03)] p=0.03	body fat at 3 month gestational age group, firstborn, time firstborn, time x bottle-feeding, season of birth, and time x season of birth<br>.
	birth - 2y, ♂♀<br>N analysis (total N): 370	yes vs no, maternal smoking, pregnancy<br>rapid weight gain [increase in weight standard deviation score >0.67 between birth and 24 months]<br>1.29(95%CI: 0.66,2.49) adjOR	
(Kwok et al. 2010)<br>prospective	China[Hong Kong] Children of 1997, 11y, ♂♀<br>N analysis (total N): 3,337(8,327)	daily paternal vs no SHS or paternal smoking, pregnancy<br>BMI z-score<br>(+) assoc. [adj mean diff.(95%CI): 0.16(0.07,0.26)]	gender, birth order, parental education, mother's place of birth, pubertal status, parental occupation, household income, breastfeeding, number of hospital admissions due to infections at 0 to 6 months
(Leary et al. 2006)<br>prospective	UK[Bristole] ALSPAC 9.9y, ♂♀<br>N analysis (total N): 3621	yes vs no, maternal smoking, pregnancy<br>BMI<br>(+) assoc. [adjβ(95%CI): 0.24(0.16,0.32)]	sex and child's age at DXA scan, maternal, partner, social, and infant feeding factors, birth weight and gestation
(Matijasevich et al. 2011)<br>prospective	Brazil[Pelotas] Pelotas 2004, 4y, ♂♀<br>N analysis (total N): 3,799	yes vs no, maternal smoking, pregnancy<br>BMI z-score<br>(+) assoc. [adjβ(95%CI): 0.19(0.12,0.26)]	income, marital status, schooling, age, skin color, parity, height, BMI, pregnancy duration, paternal smoking
	Brazil[Pelotas] Pelotas 1993, 4y, ♂♀<br>N analysis (total N): 1,450	yes vs no, maternal smoking, pregnancy<br>BMI z-score<br>no assoc. [adjβ(95%CI): 0.02(-0.01,0.05)]	
(Ravnborg et al. 2011)<br>cross-sectional	Denmark[Copenhagen] 19y, ♂<br>N analysis (total N): 3486 (4862)	yes vs no, maternal smoking, pregnancy<br>BMI<br>(+) assoc. [adjβ(95%CI): 0.1(0.05,0.15)]	smoking status of son
(Sowan and Stember 2000)<br>prospective	US[not reported] 14m, ♂♀<br>N analysis (total N): 433(630)	yes vs no, maternal smoking, pregnancy<br>obesity [BMI>84th based on age, gender)]<br>↑ w/maternal smoking [normalized effect size,% control: 18.9)]	unadjusted
(Steur et al. 2010)<br>prospective	Netherlands[multi-site] PIAMA 8y, ♂♀<br>N analysis (total N): 1,687	yes (≥5/d) vs no, maternal smoking, pregnancy<br>overweight [BMI based on age, gender specific)]<br>not considered an independent predictor of risk of	unadjusted (variables were evaluated for potential inclusion in a prediction model using stepwise

Supplemental Material Table S1 cont. Human studies on maternal smoking during pregnancy and findings related to growth in children

Reference	Cohort Description	Outcome	Adjustment Factors
		overweight (data not shown)	model selection)
(Suzuki et al. 2010)<br>prospective	Japan[Koshu City] 9-10y,<br>♂<br>N analysis: 658	current vs none/former, maternal smoking, pregnancy<br>BMI z-score<br>↑ w/maternal smoking [normalized effect size,% control(95%CI): 266.7(210.6,322.7)]	unadjusted
		BMI<br>↑ w/maternal smoking [normalized effect size,% control(95%CI): 12.1(4.9,19.2)]	
	♀<br>N analysis: 616	BMI<br>no difference [normalized effect size,% control(95%CI): 0(-7.3,7.3)]	
		BMI z-score<br>no difference [normalized effect size,% control(95%CI): -200(378.4,-778.4)]	
(Syme et al. 2010)<br>cross-sectional	Canada[Quebec] late puberty, 15-18y, ♂♀<br>N analysis (total N): 341(508)	yes vs no, maternal smoking, pregnancy<br>body weight [kg]<br>↑ w/maternal smoking [adj mean diff.(95%CI): 4.6(1.8,7.4)]	gender, birth weight, length of breastfeeding
(Wen et al. 2011)<br>prospective	US[multi-site] CPP 7y, ♂♀<br>N analysis (total N): 27625 (30,461)	yes, heavy (≥20/d) vs no, maternal smoking, pregnancy<br>BMI<br>no difference [adj mean diff.(95%CI): 0.28(-0.12,0.68)]	maternal age at pregnancy, race, marital status, family SES, parity, gestational age, child's gender

Abbreviations: adjβ – adjusted β coefficient; assoc. – association; ALSPAC – Avon Longitudinal Study of Parents and Children; BMI – body mass index; CPP – Collaborative Perinatal Project; DXA – dual-emission X-ray absorptiometry; Gen R – Generation R study; HOYVS – Health of Young Victorians study; KiGGS – German Health Interview and Examination Survey for Children and Adolescents; MAS-90 – German Multicenter Allergy Study; MDD – major depressive disorder; PIAMA – Prevention and Incidence of Asthma and Mite Allergy; SES – socioeconomic status; Δ – change

Supplemental Material Table S2. Human studies on maternal smoking during pregnancy and findings related to adiposity in children

Reference	Cohort Description	Outcome	Adjustment Factors
(Durmus et al. 2011) prospective	Netherlands[Gen R] 2y, ♂♀ N analysis (total N): 659(907)	yes (>5/d) vs no, maternal smoking, pregnancy fat, total subcutaneous [diagnostic: sum of skinfold thickness (mm) for biceps, triceps, suprailiacal and subscapular] (+) assoc. [adjβ(95%CI): 2.67(0.2,5.14)]	child's age at visit, sex, maternal education, maternal height and weight, breastfeeding ever, current height and observer
	Netherlands[Gen R] 2y, ♂♀ N analysis: 660	fat, central subcutaneous [diagnostic: sum of skinfold thickness (mm) for suprailiacal and subscapular] no assoc. [adjβ(95%CI): 1.21(-0.04,2.46)]	
	Netherlands[Gen R] 2y, ♂♀ N analysis: 672	fat, peripheral subcutaneous [diagnostic: sum of skinfold thickness (mm) for biceps and triceps] no assoc. [adjβ(95%CI): 1.46(-0.08,3.01)]	
(Leary et al. 2006) prospective	UK[Bristole] ALSPAC 9.9y, ♂♀ N analysis: 3621	yes vs no, maternal smoking, pregnancy total lean mass standard deviation score [diagnostic: DXA] (+) assoc. [adjβ(95%CI): 0.1(0.05,0.15)]	sex and child's age at DXA scan, maternal, partner, social, and infant feeding factors, birth weight and gestation
		total fat standard deviation score [diagnostic: DXA] (+) assoc. [adjβ(95%CI): 0.19(0.12,0.26)]	
		truncal fat standard deviation score [diagnostic: DXA and automatic region of interest that included chest, abdomen, and pelvis] no assoc. [adjβ(95%CI): 0.02(-0.01,0.05)]	
(Silva et al. 2010) prospective	Brazil[Ribeirao] Preto, 23-25y N analysis (total N): NR (2,063) ♀	yes vs no, maternal smoking, pregnancy adiposity [diagnostic: BMI, waist circumference, sum of triceps and subscapular skinfolds] (+)assoc. [adjβ: 0.06(p=0.056)]	unadjusted (variables were evaluated for potential inclusion in a structural equation model using adiposity as latent response variable)
	♂	adiposity [diagnostic: BMI, waist circumference, sum of triceps and subscapular skinfolds] no assoc. [adjβ: 0.004(p=0.915)]	
(Syme et al. 2010) cross-sectional	Canada[Quebec] late puberty, 15-18y, ♂♀ N analysis (total N): 341(508)	yes vs no, maternal smoking, pregnancy fat, total body [diagnostic: multifrequency bioimpedance analysis, kg] ↑ w/maternal smoking [adj mean diff.(95%CI): 0.1(0.04,0.15)]	gender, birth weight, length of breastfeeding
		fat, subcutaneous [diagnostic: MRI (mm^3)] ↑ w/maternal smoking [adj mean diff.(95%CI): 0.11(0.04,0.19)]	
		fat, intra-abdominal [diagnostic: MRI (mm^3)]	

Reference	Cohort Description	Outcome	Adjustment Factors
		↑ w/maternal smoking [adj mean diff.(95%CI): 0.14(0.06,0.22)]	
(Vik et al. 1996) prospective	Norway/Sweden[Bergan/Uppsala] ~5y, ♂♀ N analysis (total N): 343(530)	yes (time of conception) vs no, maternal smoking, pregnancy skinfold, triceps [mm] ↑ w/maternal smoking [normalized effect size,% control(95%CI): 7(1.3,12.7)]	unadjusted
	N analysis: 340	skinfold, subscapular [mm] ↑ w/maternal smoking [normalized effect size,% control(95%CI): 14.5(4.8,24.3)]	
	N analysis: 362	ponderal index [diagnostic: weight (g) x 100/length (cm^3)] ↑ w/maternal smoking [normalized effect size,% control(95%CI): 2.9(0.9,4.9)]	
	N analysis: 363	mid-abdominal diameter/weight [mm/g] no difference [normalized effect size,% control(95%CI): 2(-1.2,5.3)]	
	N analysis: 358	arm circumference [cm] no difference [normalized effect size,% control(95%CI): 1.1(-0.7,2.9)]	
(von Schnurbein et al. 2011) cross-sectional	Germany[Ulm] URMEL-ICE 7y, ♂♀ N analysis: 706	yes vs no, maternal smoking, pregnancy intra-abdominal fat [diagnostic: sonographically measured intra-abdominal depth (mm)] no assoc. [adjβ(SD): 1.3(± 0.9)]	significant factors from bivariate analysis (birth weight, SGA, weight incrase, rapid weight gain, breast feeding, parental education, parental BMI, migration from at least 1 parent
	Germany[Ulm] URMEL-ICE 7y, ♂ N analysis: 372	intra-abdominal fat [diagnostic: sonographically measured intra-abdominal depth (mm)] no assoc. [adjβ(SD): 0.5(± 1.1)]	
	Germany[Ulm] URMEL-ICE 7y, ♀ N analysis: 334	intra-abdominal fat [diagnostic: sonographically measured intra-abdominal depth (mm)] no assoc. [adjβ(SD): 2.4(± 1.3),p=0.064]	

Abbreviations: adjβ – adjusted β coefficient; adj mean diff. – adjusted mean difference; assoc. – association; BMI – body mass index; intra-abdom. – intra-abdominal; Gen R – Generation R study; NR – not reported; SD – standard deviation; SGA – small gestational age; URMEL-ICE – Ulm Research on Metabolism, Exercise and Lifestyle Intervention in Children; w/maternal smoking

Supplemental Material Table S2 cont.. Human studies on maternal smoking during pregnancy and findings related to adiposity in children

Supplemental Material Figure S2. Nicotinic receptor targets in ToxCast™

- Nicotine was not tested in Phase 1 of EPA's ToxCast™[a] initiative, but the assay battery does include an assay for nicotinic acetylcholine receptor assay (NVS_LGIC_hNNR_NBungSens)
- Some ToxCast™ Phase 1 compounds interact with this receptor target. Data are presented as active concentration 50%.
- Additional details of the assay are available at

Nicotine
CASRN: 54-11-5
$C_{10}H_{14}N_2$ (MW 162.23)

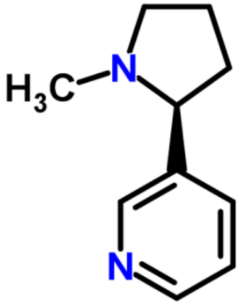

**Chemical**	**CASRN**	**NVS_LGIC_hNNR_ NBungSens (µM)**
Thiacloprid	111988-49-9	4.9
Acetamiprid	135410-20-7	5.7
Imidacloprid	138261-41-3	9.7
Cyazofamid	120116-88-3	26.0
Clothianidin	210880-92-5	30.0
Mepiquat chloride	24307-26-4	35.0

Use: Alkaloid found in the nightshade family of plants (Solanaceae) that constitutes approximately 0.6–3.0% of dry weight of tobacco. Considered the main factor responsible for dependence forming properties for tobacco use
Mechanism: binds to nicotinic acetylcholine receptors (nAChRs)

[a] ToxCast™ is EPA's contribution to Tox21, a collaborative program between the EPA, NIEHS/NTP, NIH/NCGC, and FDA designed to research, develop, validate and translate innovative chemical testing methods that characterize toxicity pathways.

References

Beyerlein A, Toschke AM, Schaffrath Rosario A, von Kries R. 2011. Risk factors for obesity: further evidence for stronger effects on overweight children and adolescents compared to normal-weight subjects. PLoS One 6(1):e15739.

Beyerlein A, Toschke AM, von Kries R. 2010. Risk factors for childhood overweight: shift of the mean body mass index and shift of the upper percentiles: results from a cross-sectional study. Int J Obes (Lond) 34(4):642-648.

Durmus B, Ay L, Hokken-Koelega AC, Raat H, Hofman A, Steegers EA, et al. 2011. Maternal smoking during pregnancy and subcutaneous fat mass in early childhood. The Generation R Study. Eur J Epidemiol 26(4):295-304.

Goldani MZ, Haeffner LS, Agranonik M, Barbieri MA, Bettiol H, Silva AA. 2007. Do early life factors influence body mass index in adolescents? Braz J Med Biol Res 40(9):1231-1236.

Griffiths LJ, Hawkins SS, Cole TJ, Dezateux C. 2010. Risk factors for rapid weight gain in preschool children: findings from a UK-wide prospective study. Int J Obes (Lond) 34(4):624-632.

Hesketh K, Carlin J, Wake M, Crawford D. 2009. Predictors of body mass index change in Australian primary school children. Int J Pediatr Obes 4(1):45-53.

Hill SY, Shen S, Locke Wellman J, Rickin E, Lowers L. 2005. Offspring from families at high risk for alcohol dependence: increased body mass index in association with prenatal exposure to cigarettes but not alcohol. Psychiatry Res 135(3):203-216.

Iliadou AN, Koupil I, Villamor E, Altman D, Hultman C, Langstrom N, et al. 2010. Familial factors confound the association between maternal smoking during pregnancy and young adult offspring overweight. Int J Epidemiol 39(5):1193-1202.

Kanellopoulos TA, Varvarigou AA, Karatza AA, Beratis NG. 2007. Course of growth during the first 6 years in children exposed in utero to tobacco smoke. Eur J Pediatr 166(7):685-692.

Karaolis-Danckert N, Buyken AE, Kulig M, Kroke A, Forster J, Kamin W, et al. 2008. How pre- and postnatal risk factors modify the effect of rapid weight gain in infancy and early childhood on subsequent fat mass development: results from the Multicenter Allergy Study 90. Am J Clin Nutr 87(5):1356-1364.

Kwok MK, Schooling CM, Lam TH, Leung GM. 2010. Paternal smoking and childhood overweight: evidence from the Hong Kong "Children of 1997". Pediatrics 126(1):e46-56.

Leary SD, Smith GD, Rogers IS, Reilly JJ, Wells JC, Ness AR. 2006. Smoking during pregnancy and offspring fat and lean mass in childhood. Obesity (Silver Spring) 14(12):2284-2293.

Matijasevich A, Brion MJ, Menezes AM, Barros AJ, Santos IS, Barros FC. 2011. Maternal smoking during pregnancy and offspring growth in childhood: 1993 and 2004 Pelotas cohort studies. Arch Dis Child 96(6):519-525.

Ravnborg TL, Jensen TK, Andersson AM, Toppari J, Skakkebaek NE, Jorgensen N. 2011. Prenatal and adult exposures to smoking are associated with adverse effects on reproductive hormones, semen quality, final height and body mass index. Hum Reprod 26(5):1000-1011.

Silva AA, Vasconcelos AG, Bettiol H, Barbieri MA. 2010. Socioeconomic status, birth weight, maternal smoking during pregnancy and adiposity in early adult life: an analysis using structural equation modeling. Cad Saude Publica 26(1):15-29.

Sowan NA, Stember ML. 2000. Effect of maternal prenatal smoking on infant growth and development of obesity. J Perinat Educ 9(3):22-29.

Steur M, Smit HA, Schipper CM, Scholtens S, Kerkhof M, de Jongste JC, et al. 2010. Predicting the risk of newborn children to become overweight later in childhood: The PIAMA birth cohort study. Int J Pediatr Obes.

Suzuki K, Kondo N, Sato M, Tanaka T, Ando D, Yamagata Z. 2010. Gender differences in the association between maternal smoking during pregnancy and childhood growth trajectories: multilevel analysis. Int J Obes (Lond).

Syme C, Abrahamowicz M, Mahboubi A, Leonard GT, Perron M, Richer L, et al. 2010. Prenatal exposure to maternal cigarette smoking and accumulation of intra-abdominal fat during adolescence. Obesity (Silver Spring) 18(5):1021-1025.

Vik T, Jacobsen G, Vatten L, Bakketeig LS. 1996. Pre- and post-natal growth in children of women who smoked in pregnancy. Early Hum Dev 45(3):245-255.

von Schnurbein J, Klenk J, Galm C, Berg S, Gottmann P, Steinacker JM, et al. 2011. Reference values and early determinants of intra-abdominal fat mass in primary school children. Horm Res Paediatr 75(6):412-422.

Wen X, Triche EW, Hogan JW, Shenassa ED, Buka SL. 2011. Prenatal factors for childhood blood pressure mediated by intrauterine and/or childhood growth? Pediatrics 127(3):e713-721.
